# Investigation of Peppermint (*Mentha piperita*) Extract on the Inhibition of Biofilm Formation by *Acinetobacter baumannii* Strains Isolated from Clinical Samples

**DOI:** 10.5812/ijpr-160772

**Published:** 2025-06-29

**Authors:** Zahra Majidi Fard, Negin Irani Mavi, Shokoofeh Akbari, Yousef Erfani, Sanaz Eghtedari

**Affiliations:** 1 Department of Biology, Faculty of Convergent Sciences and Technologies, Islamic Azad University, Tehran, Iran; 2 Department of Biology, Faculty of Basic Sciences, Central Tehran Branch, Islamic Azad University, Tehran, Iran; 3 Department of Medical Laboratory Sciences, School of Allied Medical Sciences, Tehran University of Medical Sciences, Tehran, Iran; 4 Cellular and Molecular Research Center, Iran University of Medical Sciences, Tehran, Iran

**Keywords:** *Acinetobacter baumannii*, Biofilm, *Mentha piperita*, Peppermint, PCR

## Abstract

**Background:**

*Acinetobacter baumannii* is a hospital-acquired opportunistic pathogen, with biofilm formation playing a crucial role in its multidrug resistance. Given the rise in antibiotic resistance, herbal medicines, including peppermint (*Mentha piperita*), have gained attention for their potential antibacterial properties.

**Objectives:**

This study aimed to evaluate the inhibitory effects of peppermint extract on biofilm formation in *A. baumannii* strains isolated from clinical samples.

**Methods:**

A total of 25 *A. baumannii* strains were isolated from clinical samples at the Faculty of Allied Medical Sciences, Tehran. Their biofilm-forming ability and antibiotic susceptibility against nine antibiotics were assessed using the disk diffusion method. The antibacterial activity of peppermint extract was evaluated by well diffusion, with its minimum inhibitory concentration (MIC) and minimum bactericidal concentration (MBC) compared to ciprofloxacin. Synergistic effects between the extract and ciprofloxacin were analyzed, followed by a time-kill assay. Polymerase chain reaction (PCR) was used to detect the presence of *PgaA* and *AbaI* genes linked to biofilm formation.

**Results:**

The study found that 88% of *A. baumannii* strains exhibited strong biofilm formation. Peppermint extract effectively inhibited biofilm formation, with MIC values ranging from 1.5 to 6 mg/mL (mean MIC: 3.75 ± 1.38 mg/mL) and MBC values equivalent to their respective MIC concentrations. For ciprofloxacin, the MIC for all samples was greater than 2048 mg/mL. No significant synergistic effect was observed between peppermint extract and ciprofloxacin. Both *PgaA* (involved in biofilm matrix synthesis) and *AbaI* (quorum sensing-related autoinducer synthase) genes were present in all tested strains.

**Conclusions:**

Peppermint extract demonstrates biofilm-inhibitory properties against *A. baumannii*, suggesting its potential as an alternative therapeutic approach for combating biofilm-associated infections.

## 1. Background

*Acinetobacter baumannii* is an aerobic, non-glucose-fermenting, gram-negative bacillus widely found in the environment (1). It causes various infections, primarily ventilator-associated pneumonia and bloodstream infections, with mortality rates reaching 35%. Its spread is often linked to multidrug resistance, including strains resistant to all available antibiotics (1).

Over recent decades, *A. baumannii* has developed resistance to many antimicrobials, including broad-spectrum cephalosporins, penicillins, fluoroquinolones, and aminoglycosides. A key virulence factor is biofilm formation, regulated by specific genes. Biofilms — three-dimensional bacterial communities encased in an extracellular matrix — significantly enhance resistance to antimicrobial agents (2, 3). Biofilm formation complicates treatment and facilitates transmission, contributing to hospital outbreaks. Surfaces like heart valves, prosthetic joints, ventilators, and catheters provide ideal conditions for biofilms. Clinical isolates show greater biofilm-forming ability on inert surfaces than non-clinical ones, promoting persistent hospital infections (2, 4).

Biofilms also protect *A. baumannii* from the immune system and treatments, increasing antibiotic tolerance up to a thousand-fold. Within biofilms, bacteria more readily acquire resistance through horizontal gene transfer, enhancing survival (5).

Medicinal plants have been used for centuries due to their biological effects. World Health Organization (WHO) reports that 80% of people in developed countries use them for treatment (6). Peppermint (*Mentha piperita* L) is known for antimicrobial and anticancer properties. Studies confirm its antibacterial effects, effective against both gram-positive and gram-negative bacteria (7).

Genes like *AbaI* and *PgaA* regulate *A. baumannii* biofilms. *AbaI* encodes an enzyme for quorum sensing, influencing biofilm development, while *PgaA*, part of the *PgaABCD* locus, is involved in synthesizing PNAG, a key biofilm component. Though detection of these genes helps assess biofilm potential, it doesn't always reflect their expression or biofilm strength. Understanding these genes is vital for developing anti-biofilm therapies (2, 8).

## 2. Objectives

This study aimed to investigate the inhibitory effect of peppermint plant extract on biofilm formation by *A. baumannii*.

## 3. Methods

### 3.1. Clinical Sample Collection Period

Clinical samples were collected over a 12-month period from April 2023 to March 2024 from patients admitted to various units of a hospital in Tehran. A total of 500 clinical specimens were obtained, including respiratory secretions, wound exudates, blood cultures, and urinary samples. The samples were collected as part of routine diagnostic procedures and were transferred to the microbiology laboratory within 2 hours of collection under appropriate transport conditions. All samples underwent standard microbiological processing for the isolation and identification of potential pathogens, with a specific focus on identifying *A. baumannii* isolates.

### 3.2. Collection and Preparation of the Plant and Extraction

Fresh and dried peppermint leaves were obtained from an herbal shop and authenticated by the Department of Plant Systematics, Shahid Beheshti University, Tehran. For extraction, 50 g of dried, crushed leaves were soaked in 450 mL ethanol (total 500 mL) for two days. The mixture was filtered, and ethanol was evaporated naturally in an open tray to yield a dried powder. The extract was weighed and reconstituted in ethanol as needed for experiments.

### 3.3. Chemical Characterization of Peppermint Extract

Following extraction, the chemical characterization of peppermint extract was conducted to identify and quantify its main bioactive components. Gas chromatography-mass spectrometry (GC-MS) analysis was performed using an Agilent 7890B GC system with a 5977A mass selective detector. The extract, diluted in methanol (1:100), was injected (1 μL) into an HP-5MS capillary column (30 m × 0.25 mm, 0.25 μm film).

The oven temperature was set to 60°C (2 min), increased to 240°C at 5°C/min, and held for 10 min. Helium served as the carrier gas (1 mL/min). The MS was operated in electron impact mode (70 eV) with an ion source temperature of 230°C. Compounds were identified by comparing mass spectra with the NIST library (v2.0) and matching retention indices to literature data.

High-performance liquid chromatography (HPLC) was also used to quantify major phenolic compounds. Analyses were carried out with a Shimadzu LC-20AD system and SPD-M20A diode array detector on a C18 column (250 mm × 4.6 mm, 5 μm) at 30°C. The mobile phase included 0.1% formic acid in water (A) and acetonitrile (B), with gradient elution. Flow rate was 1 mL/min, and detection was done at 280 nm. Quantification used standard curves for menthol, menthone, and rosmarinic acid (9).

### 3.4. Disk Diffusion Agar Test Method

The test followed Clinical and Laboratory Standards Institute (CLSI) guidelines. Mueller-Hinton agar plate (Merck, Germany) was prepared with a pH of 7.2 - 7.4. A standardized microbial suspension was spread evenly on the agar using a sterile cotton swab (lawn culture method). After 15 minutes at room temperature, nine antibiotic discs, also at room temperature, were placed on the agar with at least 2.5 cm spacing between discs and plate edges. Plates were incubated at 37°C for 18 - 24 hours. Inhibition zone diameters around each disc were then measured and recorded.

### 3.5. Determination of the Effectiveness of Peppermint Extract by Well Diffusion Method

A suspension of *A. baumannii* equivalent to a 0.5 McFarland standard was prepared in physiological saline and inoculated onto Mueller-Hinton agar plates. Four wells were made in the agar using a sterile punch. Peppermint extract at concentrations of 3.1, 6.25, 12.5, and 25 mg/mL was added to the wells. Plates were incubated at 37°C for 24 hours, after which inhibition zone diameters were measured and recorded.

### 3.6. Determination of Minimum Inhibitory Concentration of Peppermint Extract and Ciprofloxacin Antibiotic

The minimum inhibitory concentration (MIC) was determined using the serial microdilution broth method in a 96-well microplate. Each well received 100 µL of Mueller-Hinton broth, followed by serial dilutions of test compounds: Peppermint extract (starting at 12 mg/mL) and ciprofloxacin (starting at 2048 mg/mL). Then, 100 µL of a standardized bacterial suspension (10⁶ CFU/mL) was added to each well. Positive controls (broth + solvent) and negative controls (broth + solvent + bacteria) were included. Plates were incubated at 37°C for 24 hours, and bacterial growth was then assessed.

### 3.7. Ranking Method for Antibiotic Resistance Analysis

Antibiotic susceptibility of *A. baumannii* isolates was assessed following CLSI guidelines (M100-S30, 2024). Inhibition zone diameters were measured and classified as resistant (R), intermediate (I), or susceptible (S) based on CLSI breakpoints for *Acinetobacter* species (10).

Antibiotics were ranked by the percentage of resistant isolates, with higher resistance ranked first. When resistance percentages were equal, clinical importance and frequency of use (per institutional protocols and MDR guidelines) were considered.

Antibiotics were also grouped into classes (e.g., carbapenems, fluoroquinolones, aminoglycosides, polymyxins) to assess class-specific resistance patterns. The Multiple Antibiotic Resistance (MAR) Index was calculated for each isolate using the formula: MAR = Number of antibiotics resisted/Total antibiotics tested.

Isolates with MAR ≥ 0.2 were considered from high-risk sources with frequent antibiotic exposure.

This systematic ranking allowed for a comprehensive evaluation of resistance profiles and identification of effective treatment options, while also highlighting concerning resistance trends.

### 3.8. Determination of Minimum Bactericidal Concentration of Peppermint Extract and Ciprofloxacin Antibiotic

Ten microliters from the wells where bacterial growth was inhibited (the wells after the MIC wells) were transferred onto Mueller-Hinton agar plates and incubated at 37°C for 24 hours. After the incubation period, the plates were examined for bacterial growth. The last concentration in which no colony growth occurred was identified as the MBC.

### 3.9. Evaluation of Acinetobacter baumannii Biofilm Formation Inhibition

A TSB medium with 2% glucose was prepared, and *A. baumannii* suspensions were adjusted to 0.5 McFarland turbidity. A volume of 200 µL of the suspension was added to each well of a 96-well plate, incubated at 37°C for 24 h. Three wells containing only media (no bacteria) served as negative controls to determine the optical density cut-off (ODc) for biofilm classification.

After incubation, the medium was discarded and wells were washed twice with 200 µL PBS. Wells were fixed with 200 µL of absolute methanol for 10 minutes, stained with 200 µL of 1% crystal violet for 5 minutes, and then rinsed with distilled water. Finally, 200 µL of 33% glacial acetic acid was added to each well to solubilize the stain.

Absorbance was measured at 640 nm using an ELISA reader. While 590 - 595 nm is standard, 640 nm was used due to equipment constraints; previous studies confirm its validity for ODc-based classification. Biofilm production levels were categorized as shown in Table 1 (11, 12).

**Table 1.** Biofilm Production and Average Optical Density Classification ^a^

Biofilm Production	Average OD
**Negative**	OD ≤ ODc
**Weak**	ODc ≤ OD ≤ 2 × ODc
**Moderate**	2 × ODc ≤ OD ≤ 4 × ODc
**Strong**	4 × ODc < OD

^a^ ODc: Optical density cut-off value calculated as the mean OD of negative control wells (no bacterial growth) plus three standard deviations. This value is used as a baseline to categorize biofilm formation intensity.

In the initial method, biofilm formation by *A. baumannii* was assessed without the addition of any extract. To evaluate the potential effect of peppermint extract on biofilm formation, 200 µL of the extract at its MIC was added to the bacterial suspension in TSB medium supplemented with 2% glucose, after adjustment to 0.5 McFarland turbidity. The biofilm quantification procedure was then repeated as previously described.

In a separate experiment, 200 µL of the extract at 2MIC was added under the same conditions, and the steps were repeated accordingly. Finally, the biofilm classification results from the three experimental conditions (control, MIC, and 2MIC) were compared to assess the impact of the peppermint extract on biofilm formation.

### 3.10. Time Kill Method

One of the *A. baumannii* strains was selected, and a 0.5 McFarland suspension was prepared. The suspension was then divided into two groups: The control group, which received no treatment, and the treatment group, to which the MIC concentration of peppermint extract was added. Sampling was performed at 0, 2, 4, 6, 8, and 24 hours, and colony counts were conducted for each time point.

### 3.11. Evaluation of the Combined and Synergistic Effect of Peppermint Extract and Ciprofloxacin Antibiotic by the Checkerboard Method

The concentration range of the drugs used in the checkerboard method was the same as that used in the broth microdilution method. Fifty µL of each concentration was transferred into the wells of a sterile 96-well microtiter plate. The ciprofloxacin antibiotic concentration was equal to the MIC concentration of the peppermint extract. The ciprofloxacin concentrations ranged from 2048 to 16 mg/mL, while the peppermint extract concentrations ranged from 3 to 0.0234375 mg/mL.

First, 50 µL of ciprofloxacin antibiotic was added horizontally into the wells of the microtiter plate. Then, 50 µL of peppermint extract was added vertically into the wells. Following this, 100 µL of a bacterial suspension (equivalent to 10^6^ CFU) was added to all the wells. The microtiter plates were incubated at 37°C for 18 to 24 hours. After this incubation period, the MIC values were read again.

### 3.12. Detection of Biofilm-Associated Genes

Polymerase chain reaction (PCR) was conducted to detect the *AbaI* and *PgaA* genes in *A. baumannii* isolates (6). Each 25 μL reaction contained 12.5 μL of 2X PCR master mix (Ampliqon, Denmark), 1 μL (10 pmol) of each primer (Table 2), 2 μL of template DNA (~50 ng), and 8.5 μL of nuclease-free water. Amplification was carried out on a Bio-Rad T100™ thermal cycler under the following conditions: Initial denaturation at 95°C for 5 min; 35 cycles of 95°C for 30 s, 58°C for 30 s, and 72°C for 45 s; with a final extension at 72°C for 7 min.

**Table 2.** Primer Size and Sequence Information Used in the Study

Genes	Product Size	Primer Forward	Primer Reverse
***AbaI***	150 bp	CCGCCTTCCTCTAGCAGTCA	AAAACCCGCAGCACGTAATAA
***PgaA***	150 bp	GCCGACGGTCGCGATAC	ATGCACATCACCAAAACGGTACT

Polymerase chain reaction products were resolved on a 1.5% agarose gel stained with 0.5 mg/mL ethidium bromide, run in 1× TAE buffer at 80 V for 60 min. A 100 bp DNA ladder (Smobio, Taiwan) was used as a size marker. The presence of 150 bp bands indicated successful amplification of the target genes. This qualitative analysis was performed to confirm the biofilm-related genetic potential of the isolates before testing the anti-biofilm effects of peppermint extract.

### 3.13. Polymerase Chain Reaction Reference Control

For PCR validation and quality control purposes, *A. baumannii* ATCC 19606 was employed as the reference strain and positive control. This well-characterized strain is known to possess both the *AbaI* and *PgaA* genes and exhibits strong biofilm-forming capabilities, making it an appropriate benchmark for our molecular analyses. The genomic DNA extracted from ATCC 19606 consistently produced clear amplification bands at the expected 150 base pair region for both target genes, confirming the specificity and efficiency of our primer design and PCR conditions (13).

### 3.14. Statistical Analysis

Statistical analyses were performed with SPSS version 25.0 (IBM Corp., Armonk, NY, USA). Experiments were done in triplicate, and results are shown as mean ± SD. One-way ANOVA with Tukey’s post-hoc test was used to analyze the inhibitory effects of peppermint extract on biofilm formation and differences in inhibition zone diameters across concentrations. For the time-kill assay, bacterial counts (CFU/mL) were log-transformed and analyzed by repeated measures ANOVA with Bonferroni correction. The distribution of biofilm strength categories was assessed by chi-square test. A P-value < 0.05 was considered statistically significant.

## 4. Results

### 4.1. Antibiotic Sensitivity Pattern of the Samples

According to the results obtained, colistin was the only antibiotic that showed sensitivity (92% sensitive). Ampicillin, with 12% sensitivity, ranked second, while all other antibiotics tested were 100% resistant (Table 3). 

**Table 3.** Antibiotic Resistance, Sensitivity, and Intermediate Sensitivity results

Antibiotics	Resistant	Intermediate	Susceptible
**Meropenem**	100	0	0
**Ciprofloxacin**	100	0	0
**Colistin**	8	0	92
**Trimethoprim-sulfamethoxazol**	100	0	0
**Peperacillin**	100	32	0
**Ampicillin-sulbactam**	56	0	12
**Ceftazidime**	100	0	0
**Gentamycin**	100	0	0
**Tetracycline**	100	0	0

### 4.2. Chemical Characterization of Peppermint Extract

The chemical profile of the peppermint extract was analyzed using GC-MS and HPLC. Gas chromatography-mass spectrometry analysis revealed the presence of several key volatile compounds. The most abundant constituents identified were:

- Menthol (31.6%)

- Menthone (22.4%)

- 1,8-Cineole (Eucalyptol) (12.8%)

- Menthyl acetate (9.3%)

- Limonene (6.7%)

- β-Caryophyllene (5.1%)

High-performance liquid chromatography quantification confirmed the presence of:

- Menthol: 19.2 mg/g dry weight

- Menthone: 13.7 mg/g dry weight

- Rosmarinic acid: 8.4 mg/g dry weight

These compounds are known for their antimicrobial, anti-inflammatory, and antioxidant properties and are likely contributors to the observed antibacterial and anti-biofilm effects of the extract.

### 4.3. Antibacterial Activity of Peppermint Extract on Acinetobacter baumannii Using the Agar Well Diffusion Method

The results indicated that peppermint extract inhibits the growth of *A. baumannii*. At a concentration of 25 mg/mL, 80% of the strains were inhibited; at a concentration of 12.5 mg/mL, 92% of the strains were inhibited; at a concentration of 6.25 mg/mL, 92% of the strains were inhibited; and at a concentration of 3.1 mg/mL, 96% of the strains showed resistance.

### 4.4. Evaluation of Minimum Inhibitory Concentration and Minimum Bactericidal Concentration of Peppermint Extract and Ciprofloxacin Against Acinetobacter baumannii

In the study of 25 *A. baumannii* strains, the MIC of peppermint extract was found to be 1.5 mg/mL for 4% (1/25) of the strains, 3 mg/mL for 68% (17/25) of the strains, and 6 mg/mL for 28% (7/25) of the strains, with a mean MIC value of 3.75 ± 1.38 mg/mL. For ciprofloxacin, the MIC for all samples (25/25, 100%) was greater than 2048 mg/mL. The minimum bactericidal concentration (MBC) values were equivalent to the respective MIC values for both peppermint extract and ciprofloxacin in all tested strains, indicating bactericidal activity at the same concentrations that inhibited growth.

### 4.5. Investigation of Biofilm Formation Inhibition of Acinetobacter baumannii by Peppermint Extract

To assess inhibition of *A. baumannii* biofilm formation, three conditions were tested. In the control group, 88% (22/25) of isolates formed strong biofilms (mean OD640 = 0.89 ± 0.21), 8% (2/25) moderate (0.56 ± 0.08), and 4% (1/25) weak (0.31 ± 0.04). At the MIC concentration of peppermint extract, 84% (21/25) remained strong biofilm producers (0.79 ± 0.18), 12% (3/25) moderate (0.52 ± 0.07), and 4% (1/25) weak (0.29 ± 0.03), with no significant reduction versus control (P = 0.087).

At 2MIC, only 20% (5/25) formed strong biofilms (0.62 ± 0.14), 16% (4/25) moderate (0.48 ± 0.06), and 64% (16/25) weak (0.24 ± 0.07), showing a highly significant reduction compared to control and MIC groups (P < 0.001).

**Figure 1.**
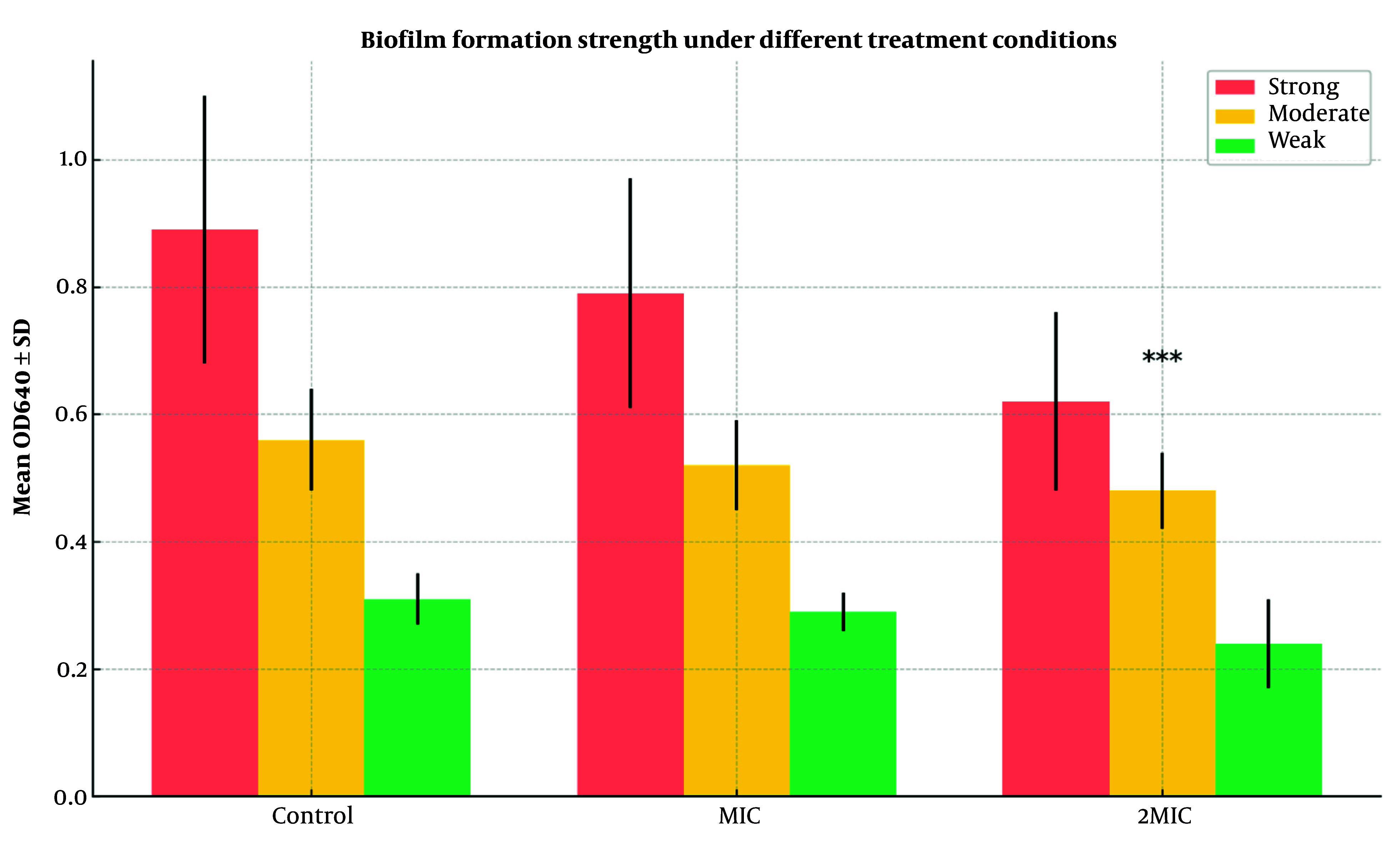
Biofilm formation strength of *Acinetobacter baumannii* isolates under different treatment conditions. The bar graph compares the distribution of biofilm production categories (strong, moderate, weak) among control, minimum inhibitory concentration (MIC), and 2MIC treatment groups. Error bars represent standard deviations of OD640 (optical density at 640 nm) values within each group. Statistical significance was assessed using chi-square and one-way ANOVA. *** P < 0.001 compared to control and MIC groups.

Figure 1 illustrates that 2MIC significantly reduced strong biofilm formation from 88% to 20% and increased weak biofilm formation from 4% to 64% (P < 0.001), indicating a potent anti- biofilm effect.

### 4.6. Results of Time-Killing Assay

#### 4.6.1. Diameter of Inhibition Zones in Agar Well Diffusion Assay

Time-kill kinetics of peppermint extract against a representative *A. baumannii* strain were assessed over 24 hours. At 0 hours, bacterial counts were 0.22 × 10⁵ CFU/mL in the control and 0.3 × 10⁴ CFU/mL in the treatment group. The control group exhibited exponential growth, reaching 0.14 × 10²¹ CFU/mL at 24 hours, while the treatment group showed a significant, time-dependent decline, with complete eradication (no detectable colonies) by 24 hours.

As shown in Table 4, fold-reduction (control CFU/treatment CFU) increased from 7.3-fold at baseline to total elimination at 24 hours, demonstrating strong bactericidal activity. Repeated measures ANOVA revealed significant differences between groups at all time points (P < 0.001), confirming the extract’s potent and time-dependent antibacterial effect.

**Table 4.** Results of the Time-Killing Assay with Fold Reduction

Hour	Control Group (CFU/mL)	Standard Strain (CFU/mL)	Fold Reduction
**0**	0.22 × 10^5^	0.3 × 10^4^	7.3
**2**	0.6 × 10^5^	0.2 × 10^4^	30.0
**4**	0.68 × 10^5^	0.14 × 10^4^	48.6
**6**	0.73 × 10^6^	0.121 × 10^2^	6 × 10^3^
**8**	0.76 × 10^7^	0.1 × 10^1^	7.6 × 10^6^
**24**	0.14 × 10^21^	0	Complete killing

The inhibition zone diameters demonstrated a clear dose-dependent response, with the highest concentration (25 mg/mL) yielding the largest zones (15.6 - 20.3 mm). Two isolates (AB-19 and AB-24) were completely resistant, showing no inhibition at any concentration. Mean inhibition zone diameters were 17.7 ± 1.5 mm at 25 mg/mL, 14.8 ± 1.3 mm at 12.5 mg/mL, 11.2 ± 1.1 mm at 6.25 mg/mL, and 7.1 ± 1.0 mm at 3.1 mg/mL. Detailed results for each isolate are provided in Table 5. 

**Table 5.** Inhibition Zone Diameters (mm) for Peppermint Extract Against *Acinetobacter baumannii* Clinical Isolates ^a^

Isolate ID	Peppermint Extract Concentration			
	25 mg/mL	12.5 mg/mL	6.25 mg/mL	3.1 mg/mL
**AB-01**	18.4 ± 0.6	15.2 ± 0.4	11.6 ± 0.5	7.2 ± 0.3
**AB-02**	17.2 ± 0.5	14.9 ± 0.6	10.8 ± 0.4	6.8 ± 0.4
**AB-03**	19.6 ± 0.4	16.3 ± 0.5	12.7 ± 0.6	8.4 ± 0.5
**AB-04**	16.8 ± 0.7	13.7 ± 0.3	10.2 ± 0.3	6.5 ± 0.3
**AB-05**	20.3 ± 0.5	17.2 ± 0.6	13.4 ± 0.5	9.1 ± 0.6
**AB-06**	17.5 ± 0.6	14.8 ± 0.4	11.3 ± 0.4	7.0 ± 0.4
**AB-07**	16.9 ± 0.3	13.5 ± 0.5	10.1 ± 0.6	6.3 ± 0.2
**AB-08**	19.8 ± 0.7	16.7 ± 0.7	12.9 ± 0.3	8.6 ± 0.5
**AB-09**	15.6 ± 0.4	12.8 ± 0.3	9.6 ± 0.4	5.8 ± 0.3
**AB-10**	18.9 ± 0.5	15.6 ± 0.6	12.1 ± 0.5	7.9 ± 0.4
**AB-11**	16.4 ± 0.6	13.2 ± 0.4	9.8 ± 0.3	6.0 ± 0.5
**AB-12**	17.8 ± 0.4	14.5 ± 0.5	11.2 ± 0.6	7.3 ± 0.3
**AB-13**	20.1 ± 0.7	17.0 ± 0.3	13.3 ± 0.4	8.9 ± 0.6
**AB-14**	18.3 ± 0.5	15.1 ± 0.6	11.7 ± 0.5	7.4 ± 0.4
**AB-15**	19.5 ± 0.6	16.4 ± 0.4	12.5 ± 0.3	8.2 ± 0.5
**AB-16**	17.1 ± 0.4	14.3 ± 0.5	10.9 ± 0.6	6.7 ± 0.3
**AB-17**	18.7 ± 0.5	15.5 ± 0.7	11.9 ± 0.4	7.6 ± 0.6
**AB-18**	16.2 ± 0.3	13.1 ± 0.4	9.7 ± 0.5	5.9 ± 0.4
**AB-19**	0	0	0	0
**AB-20**	19.2 ± 0.6	16.1 ± 0.5	12.3 ± 0.3	8.0 ± 0.5
**AB-21**	17.6 ± 0.4	14.6 ± 0.6	11.0 ± 0.5	6.9 ± 0.3
**AB-22**	18.2 ± 0.5	15.0 ± 0.3	11.5 ± 0.6	7.5 ± 0.4
**AB-23**	16.5 ± 0.7	13.4 ± 0.5	10.0 ± 0.4	6.2 ± 0.5
**AB-24**	0	0	0	0
**AB-25**	19.4 ± 0.6	16.2 ± 0.4	12.6 ± 0.5	8.3 ± 0.6
**Mean ± SD**	17.7 ± 1.5	14.8 ± 1.3	11.2 ± 1.1	7.1 ± 1.0

^a^ Values represent mean ± standard deviation of three independent experiments. Zero (0) indicates no inhibition zone observed.

One-way ANOVA confirmed significant differences across all concentrations (P < 0.001), validating the concentration-dependent antibacterial effect of peppermint extract against *A. baumannii* clinical isolates.

### 4.7. Results of Synergistic and Combined Effect of Peppermint Extract and Ciprofloxacin Antibiotic by Checkerboard Method

The interaction between peppermint extract and ciprofloxacin was assessed using the Fractional Inhibitory Concentration Index (FICI). Only 4% (1/25) of combinations showed synergy (FICI ≤ 0.5), 16% (4/25) were additive (0.5 < FICI ≤ 1.0), 4% (1/25) had an unequal response, and 76% (19/25) showed no interaction (FICI > 1.0). The mean FICI was 1.38 ± 0.47, indicating mainly indifferent interactions. These results suggest that combining peppermint extract with ciprofloxacin does not significantly improve antibacterial efficacy compared to using each alone (P = 0.72).

### 4.8. Results of AbaI and PgaA Gene Analysis

**Figure 2.**
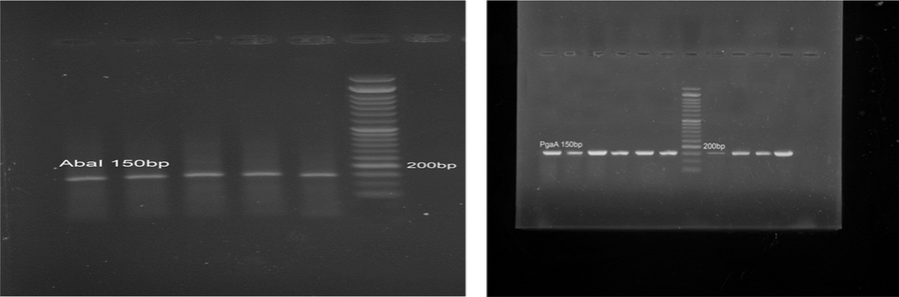
Polymerase chain reaction (PCR) gel electrophoresis results for the detection of *AbaI* and *PgaA* genes in *Acinetobacter baumannii* samples. The clear presence of a band at the 150 base pair region indicates the existence of these genes in all 25 samples examined.

Following DNA extraction, PCR was employed to detect the presence of the *AbaI* and *PgaA* genes. The resulting PCR products were then analyzed using gel electrophoresis with a Gel Documentation system. A clear band at approximately 150 base pairs confirmed the presence of the *AbaI* gene in every sample. In fact, all 25 *A. baumannii* isolates tested positive for *AbaI*. Similarly, the *PgaA* gene was detected as a 150 bp band in all samples (Figure 2), indicating its universal presence among the isolates. These findings demonstrate the consistent occurrence of both the *AbaI* and *PgaA* genes in *A. baumannii,* which could have important implications for understanding the organism’s pathogenic mechanisms.

## 5. Discussion

*Acinetobacter baumannii* is the second most common non-fermentative bacterial pathogen and a major cause of hospital-acquired infections, especially in ICUs, with mortality rates from 7.8% to 43% (7). Its ability to form biofilms — structured bacterial communities within an extracellular matrix — greatly increases resistance to antimicrobials. Biofilms are involved in at least two-thirds of bacterial infections, complicating clinical treatment due to their antibiotic resilience.

Rising antibiotic resistance highlights the need for alternative therapies. Plant-based antimicrobials are promising because many medicinal plants contain compounds that inhibit biofilm formation, boost antibiotic effectiveness, or have direct antibacterial activity. Such natural compounds may offer sustainable, effective options against biofilm-related infections.

Peppermint, from the Lamiaceae family, has anti-inflammatory, antibacterial, antiviral, and immunomodulatory properties. With increasing *A. baumannii* resistance to antibiotics, peppermint extract is a potential alternative, as its bioactive compounds might reduce resistance and inhibit biofilms (14, 15). Further studies are needed to identify the active compounds and their mechanisms.

### 5.1. Antibiotic Susceptibility Testing

In this study, antibiotic susceptibility testing was performed to assess resistance patterns among *A. baumannii* isolates. The results indicated that colistin was the only antibiotic to which all tested strains were sensitive, a finding that aligns with previous studies. For instance, Ghajavand et al. reported 92% sensitivity to colistin (16), while Smolyakov et al. observed 100% sensitivity to both colistin and ampicillin-sulbactam (17). However, our study revealed a 56% resistance rate to ampicillin-sulbactam, indicating an alarming increase in antibiotic resistance over time. These findings emphasize the need for continuous monitoring of resistance trends and the exploration of alternative treatments.

### 5.2. Antimicrobial Activity of Peppermint Extract

The agar well diffusion method demonstrated that peppermint extract effectively inhibited *A. baumannii* growth, reinforcing its potential as a natural antimicrobial agent. These findings align with previous research, such as Pramila et al., who reported significant antimicrobial activity of peppermint due to its bioactive compounds (18). Similarly, Jeyakumar et al. found that peppermint extract exhibited concentration-dependent antibacterial effects, demonstrating its efficacy against multidrug-resistant bacterial strains (19).

### 5.3. Minimum Inhibitory Concentration and Minimum Bactericidal Concentration Analysis

The MIC and MBC of peppermint extract and ciprofloxacin were assessed to determine their bacteriostatic and bactericidal effects. Peppermint extract showed MIC values between 1.5 and 6 mg/mL, while ciprofloxacin had a MIC of 2048 mg/mL, indicating high antibiotic resistance. The MBC values matched the MICs, demonstrating peppermint extract’s strong bactericidal activity against *A. baumannii*. Similar findings were reported by Hatami Pirghibi et al., who observed peppermint extract inhibiting Pseudomonas aeruginosa at similar concentrations (20). Additionally, peppermint extract’s potent antimicrobial effects against gram-positive and gram-negative bacteria are attributed to bioactive compounds like menthol and flavonoids, supporting its potential as a natural alternative against resistant strains (14, 21).

### 5.4. Biofilm Inhibition Assay

Biofilm formation is a major contributor to antibiotic resistance in *A. baumannii*, making its inhibition a key target for alternative treatments. Our study found that in the control group, 88% of strains formed strong biofilms, whereas treatment with peppermint extract at 2MIC concentration reduced strong biofilm formation to 20%, with 64% of strains exhibiting weak biofilm formation. This suggests that peppermint extract has a strong inhibitory effect on biofilm formation, which is crucial for limiting *A. baumannii* persistence in clinical settings. These findings align with previous research by Ranjbar and Farahani, who found that 70.6% of *A. baumannii* strains formed strong biofilms (22). Additionally, Sandas et al. demonstrated that plant-based extracts, including peppermint, effectively inhibited biofilm formation, supporting our observations (23).

### 5.5. Synergistic Effect of Peppermint Extract and Ciprofloxacin

The combination of peppermint extract with ciprofloxacin was assessed for possible synergistic effects, but no significant interaction was observed. This result differs from previous findings, such as Rosato et al.’s study, where plant extracts demonstrated synergistic activity with antibiotics like gentamicin (24). The absence of synergy in our study suggests that while peppermint extract is effective on its own, its combination with ciprofloxacin may not enhance antibacterial efficacy against *A. baumannii*. Future research should investigate other antibiotic combinations to determine potential synergistic effects with peppermint extract.

### 5.6. Genetic Basis for Biofilm Formation

Our study found that all tested *A. baumannii* isolates possessed both *AbaI* and *PgaA* genes, confirming their genetic capacity for biofilm formation. This aligns with the 88% of isolates showing strong biofilm formation phenotypically. Similar results were reported by Xiang et al., who linked *AbaI* expression to biofilm formation in drug-resistant *A. baumannii* (25), and Choi et al., who showed the *PgaABCD* locus is vital for producing poly-β-1-6-N-acetylglucosamine, a key biofilm matrix component (26). Although we detected these genes, future studies should use quantitative RT-PCR to assess if peppermint extract modulates their expression, which could clarify the molecular basis of its anti-biofilm effects and how natural compounds disrupt biofilm formation genetically.

### 5.7. Conclusions

This study provides strong evidence that peppermint extract exhibits inhibitory effects on *A. baumannii* growth and biofilm formation, making it a potential alternative therapy for biofilm-related infections. Given the increasing resistance of *A. baumannii* to conventional antibiotics, exploring plant-based antimicrobials represents a valuable strategy for future clinical applications. Further studies should focus on identifying the bioactive compounds responsible for these effects, understanding their mechanisms of action, and assessing their potential synergistic interactions with existing antibiotics.

## Data Availability

The dataset presented in the study is available on request from the corresponding author during submission or after publication.
